# Ethical, Legal, and Religious Aspects at the Border of Viability

**DOI:** 10.3389/fped.2019.00175

**Published:** 2019-05-08

**Authors:** Frank Oehmke, Tina Lauer, Johanna Baecker, Silke Mader, Nedim Soydan, Thomas Born, Matthias Brumhard, Reinhard Dettmeyer, Schimon Staszewski, Thomas Heinemann, Ulrika Kilian, Yasar Sarikaya, Hartmut Kress, Hans-Rudolf Tinneberg, Yasar Bilgin, Klaus-Peter Zimmer, Harald Ehrhardt

**Affiliations:** ^1^Department of Obstetrics and Gynecology, Justus-Liebig-University, Giessen, Germany; ^2^Department of General Pediatrics and Neonatology, Justus-Liebig-University, Giessen, Germany; ^3^European Foundation for the Care of Newborn Infants, Munich, Germany; ^4^Türkisch-Deutsche Gesundheitsstiftung e.V., Giessen, Germany; ^5^Clinical Pastoral Care, University Hospital of Gießen, Giessen, Germany; ^6^Ethics Delegate, Medical Management, University Hospital of Gießen, Giessen, Germany; ^7^Institute of Forensic Medicine, Giessen, Germany; ^8^General Practicioner, Langen, Germany; ^9^Chair of Ethics, Theory and History of Medicine, Philosophical-Theological University of Vallendar, Vallendar, Germany; ^10^Department of History and Cultural Studies, Giessen, Germany; ^11^Department of Social Ethics, Faculty of Protestant Theology, Bonn University, Bonn, Germany; ^12^Department of Internal Medicine III, Giessen University Hospital, Giessen, Germany

**Keywords:** preterm, border of viability, decision making, religious, ethical, legal

## Introduction to the Topic

Improvements in clinical care of the preterm infant have led to a dramatically improved outcome with respect to survival rates and survival without severe disability (1). A critical discussion of treatment results is obligatory in situations where the legal frameworks allow discussing whether (i) active resuscitation is justifiable or (ii) prolongation of therapy needs to be discontinued to prevent further suffering. Based on these data, legal conditions are periodically adapted.

The mandatory provision of active care to the preterm infant is mostly based on gestational age, which is important when survival and survival without severe impairment are considered. The increase in gestational age leads to a dramatically better outcome, especially between weeks 22 and 26. The broad variability of outcome within 1 week of gestation can be explained by the huge impact of further risk factors including birth weight, gender, completed antenatal steroids within 7 days before delivery, and antenatal growth restriction. Therefore, a stratified approach based on risk calculation is more appropriate to predict outcome (2, 3). Results from cohort studies display a wide variability of survival rates between different hospital units and countries. This cannot be solely explained by the genetic background or therapeutic options available but needs to take into account center size, treatment experience and the attitude toward provision of active care (4, 5). Similarly, attitudes toward termination of intensive care therapy in case of severe neurologic prognosis and even in the case of fatal or terminal illness display huge variability across Europe.

The newborn with congenital disorders, malformations or severe birth complications represents the second and much more diverse neonatal population where (i) initiation or (ii) discontinuation of active therapy needs to be critically considered. Generally, prediction accuracy is hampered by limitations in data availability. For selected disease entities like congenital diaphragmatic hernia, better prediction accuracy by the latest imaging techniques, data covering all relevant outcome aspects and the availability of long-term outcome facilitate parental counseling (6, 7). Providing another dimension for counseling, the potential of prenatal interventions and the latest advances in therapies with high risks for long-term health like ECMO in specialized centers need to be addressed adequately when counseling parents (8). It must be acknowledged that recent reports on newborns with restricted life expectancy, such as those with trisomy 18, display unexpected survival perspectives and successful surgical interventions with improved quality of life justifying the provision of medical therapy which was formerly excluded by the professional team (9). Even the combination of prematurity and critical congenital malformations like heart defects resulted in surprisingly high survival rates (10).

The precise prediction of long-term outcome in all populations is restricted by the fact that the consequences of acute morbidities in infants treated today on organ function in adulthood are not available but must be deducted from older patient cohorts (11). New therapeutic approaches that prove efficient in preclinical animal models raise hopes of restoring organ function in the near future (12, 13). However, these aspects cannot be considered in consultations and decisions today. Similar variations apply to the form of parental involvement in the decision process, although parental involvement by itself is accepted within clinical teams (14, 15). This is of special importance as the child surviving with functional limitations poses an enormous burden on the family. Despite the described positive effects on family cohesion and the support by relatives and friends, a handicapped infant inflicts great challenges to the stability of the marriage, the participation in social life, relationships and employment—especially for the mother (16, 17). Vice versa, insufficient parental support poses a special risk to the child with a handicap (18). Last but not least, the quality of life is judged to be much better and the functional limitations less severe by former preterm infants and their parents than by the professional team of doctors and nurses (19).

## Ethical and Legal Considerations in the Decision Process

Despite the different constitutional laws in the world, the right of all human beings to live and to get medical care is generally accepted. However, extremely preterm babies with severe and non-treatable diseases may develop a fatal prognosis, and the question of limitations in the treatment arises. On the one hand, there is the medical standard, defined by physicians, and on the other hand is the written law and decisions of courts. Of course, the parents have a right to get the information they need to make informed decisions on their child's behalf. While the constitutions and/or laws guarantee the right of the parents to care for their child, extremely preterm babies with severe diseases can have no real chance of survival, especially when born with a gestational age of <22 weeks. The situation is in discussion when born between 22 and 24 weeks. There is no obligation to treat a preterm baby when born earlier than 22 weeks (Japan, Germany, Austria, Italy), 23 weeks (USA, UK), or 24 weeks (Switzerland, Netherland, France). Other countries have similar rules (20).

A regional court in Germany decided that there is no obligation to treat a preterm baby born at 22 weeks (21). Medical guidelines and the courts ask for informed consent: if there is an agreement between parents and caregivers that intense measures will not improve the chance of survival, or pose an unacceptable burden to the child, those measures can be withheld (22). If there is no agreement, involvement of the courts is considered as a last resort (23). There are different situations:

1. Physicians want to start therapy, parents refuse to agree, partly because of seeing a long-term morbidity and suffering of their child.
2. Physicians cannot see a reason to offer therapy and prefer palliative care, parents nevertheless ask for maximal therapy.
3. Despite the decision to start therapy or not, consent must be found about resuscitation, if necessary.

If physicians offer a therapy according to the medical standard, parents cannot refuse to agree. If so, courts will regard this as misusing the right of child custody and will mandate a professional caregiver to decide in the best interest of the child. This caregiver generally will follow the decisions of the physicians. Otherwise, according to the law, the medical standard of care is what physicians have to offer based on scientific evidence (“state of the art,” “good medical practice”). But medical recommendations to offer therapy highly differ between different countries and do not necessarily reflect the complexity of the prognosis. They are mainly available for the industrialized world and some countries like Argentina, while in resource-limited countries differences and restraints in technical and personal equipment impose the establishment of general rules and recommendations (2, 24–26). If there is no medical indication for therapy due to severe diseases, this must be stated by the treating physicians as a matter of fact. Although parents ask to do more, there is no juristic obligation to follow the parents' irrational wishes.

In the same way, physicians should define those situations where withholding resuscitation is ethical—if possible, in agreement with the parents (27). This so-called “Do-not-resuscitate-order” might not be justifiable if the newborn is predicted to have >50 percent chance of survival without neurodevelopmental impairment. In contrast to physicians, the courts and lawyers are in discussion regarding whether the limit of 50 percent is acceptable. The legal principle that underlies all decisions is that of the “best interests” of the child (28). The majority perhaps prefer an obligation to resuscitate if there is a chance to live a certain time without suffering and no high risk of further resuscitations within a short period.

## Impact of the Religious Background on the Decision

Parental decision making is not only influenced by statements of the treating physician, attitudes of parents, family members and friends but also by religious attitudes. We present central aspects for each religious background based on a literature research and the general religious consent, which are summarized in Table 1.

**Table 1:** Impact of the religious background on the process of decision making.

**Religious background**	**Important aspects**
The Catholic position	Religious reason for human dignity: each human being is made in the likeness and image of God Secular ethical reason for human dignity: man is a rational being who takes responsibility for his/her actions Both concepts of human dignity result in a right to life Neither concept of human dignity demand preserving life if medical indication is lacking
The Protestant position	Basis of ethical considerations is the recognition of the child as an individual Central is the child's perspective for future life, welfare and development The child's right to live and to protect his health does not prohibit limitations of medical therapy if indicated
The Jewish position	Each human being has an indefinite value in Judaism Jewish ethics favors a casuistic and inductive approach based on the medical situation Life begins with the first independent breath and even very high risks for serious diseases constitute no convincing argument to omit life-saving activities
The Islamic position	Five fundamental principles must be followed in the decision process Comprehensive intention to choose the best medical option to serve the welfare of the child Termination of therapy is a legitimate option even though preserving life is a central Islamic concept A spiritual care giver should help the parents through this decision

### The Catholic Position

In view of a Catholic position, the right to life of each individual is indispensable and cannot be subject to assessment or weighing against other goods or desires. Each human being is considered not to be a product of chance but to be desired in God's own likeness. As being made in the likeness and image of God man represents dignity which forbids the taking his or her life by other human beings (29, 30). In a secular approach of ethics, the notion of human dignity is founded on the conviction that man is a rational being, leading to similar consequences (31). It is the special dignity of each human being which needs to be respected and, if it is in danger of being violated, to be protected, as laid down, for example, in Article 1 of the German basic law (32). Human dignity results in the right to life which forbids weighing of the life of an unborn or newborn child in the form of a value judgement. Against this background, abortion may be justifiable only if the life of the mother is acutely threatened by continuing the pregnancy. However, human dignity itself may also demand to define the limits of medical therapy. If there is no medical indication to continue life-supporting therapy, there is no moral obligation or religious duty to preserve life by every means possible. Rather, a palliative approach including the consequent reduction of pain is justified. In addition to the care for the dying child, the care for the living—the parents—is of great importance as they are suffering from the situation and the decisions to be made. It may be a chance of religion to open, with due restraint and meant as an offer, a context of understanding and acceptance to the parents.

### The Protestant View

Protestantism does not have an ecclesiastical magisterium and no moral prescriptions. With regards to medical ethics, Protestantism has known the same plurality of opinions as society at large. This concerns issues such as abortion or questions regarding euthanasia (33).

In the field of neonatology, the basis of ethical deliberations is the recognition that a child is an individual human being from birth (34). It is endowed with the right to life as well as the right to health protection and healthcare provision. In some circumstances it is doubtful whether premature infants should be kept alive or whether they should be given palliative care and be allowed to die. It falls to the parents to take such decisions on behalf. However, it should be noted that children are not the “property” of their parents but rather that they are independent subjects. When parents are put into a position in which they are responsible for deciding on behalf of their infant whether or not to continue a given therapy or cease treatment, they should take into account the perspectives of the child's future life, its welfare and its development opportunities.

In neonatal borderline situations a physician's conscience is also challenged. Physicians must distinguish between their professional medical conscience and their personal moral convictions. They must not seek to influence parents with their own private convictions, be they religious, agnostic, or atheist.

In case of decision conflicts, parents may possibly hope for help from their respective religion. For this reason, they should have access to a pastor whom they trust. From a Protestant perspective it could be sensible to offer parents the possibility of having their child baptized. As a religious symbol, baptism expresses that, despite all medical difficulties, the infant possesses its own dignity and that it is an equal member of human society and the Christian community. It is important that parents not only have access to religious pastoral care, but that psychosocial counseling is also made available to them.

### Important Aspects of Judaism

Jewish ethics arose more than 3,000 years ago from the laws of the Torah (the first five books of the Jewish Bible), the Talmud (a primary source of Jewish religious law) and other fundamental writings. Jewish tradition results from examples; it is a casuistic approach to situations occurring in daily life. Changing conditions of modern life and medical situations were and are again and again opening up for controversial discussion. They follow an inductive approach where each individual case is analyzed based on these sources and precedents to reach a decision.

In Judaism, each human being has an infinite value. Therefore, nearly all religious rules can be suspended in order to save a human life. However, this requires situation-specific decisions. Consequentially, each patient must be seen as an individual and all necessary measures have to be taken for healing and for relief in case of incurable diseases.

These rules apply equally to adult patients and severely ill as well as viable newborn and preterm infants, but not equally to the unborn child. Until birth it is considered as part of the mother and not as an own personality that enjoys the same substantial protective rights, as the life of the mother always has the preference.

But which status and function has the incubator in the context of neonatal medicine in Jewish medical ethics? On the one hand it is a usual medical device after birth. Within the situation of extremely preterm delivery and medical emergency situations the case is different.

These highly premature babies without sufficient breathing need intensive care medicine including artificial ventilation to have a chance to survive. According to Jewish law life begins with the first independent breath.

In such situations, the incubator can be seen as a time-limited substitute of the mother until the preterm displays signs of maturity according to the corresponding Halachic (the Jewish religious law) implications and consequences. Whether and which medical steps are taken depends on the chances of survival. Even very high risks of serious subsequent diseases or disability as well as the foreseeable burden for parents and the family do not constitute convincing arguments to omit life-saving activities according to the Responsa of Rabbi Eliezer Waldenberg (35, 36).

### The Islamic Perspective

Within the discussion of neonatal care at the border of viability there are five principles that must be followed by an Islamic perspective. These fundamental principles (maqāsid) serve the benefits of mankind and should be in an Islamic perspective the base of all decisions (37). First, the comprehensive will to protect the descendants, life, human dignity and the well-being of the infant. Second, the decisions about therapy must be well-grounded in the expertise of the physician, intellect and the collective consultation of physician and parents. Through this decision-making process the action of the parents corresponds with the responsibility to find the best solution for the infant. This principle is also in the realm of the third one, which is the comprehensive intention to choose the best medical option. The expected distress of the infant is an important point of judgement and should be as small as possible (38). Just in this way it is possible to follow the highest principle of the Quran: to serve the welfare of mankind (39).

In addition, if the distress of the infant is not rational, it is in Islam a legitimate option to end a therapy, even though the effort to save a life is one of the most important Islamic concepts. But there is as well the principle that emergencies set aside interdictions (38). Through this principle parents and physicians shall be able to end a therapy without being blamed in keeping focus on what is best for the infant.

A decision about the ending of therapy and therefore about the time of death of one's own child is not an easy one. It is a challenging task to be rational in a highly emotional situation. To provide support in such a situation, it is necessary to have spiritual care around that which gives the parents hope and consolation. An Islamic spiritual caregiver shall help the parents to deal with their fears and to take away or reduce the religious worries of the parents. That is important, because parents may feel guilty and as if they are committing a sin, because they do not know about all Islamic principles.

## Summary and Conclusions

Guidelines toward resuscitation or continuation of active care at the border of viability and in case of severe and prognosis-limiting disorders are mostly restricted to industrialized countries and display substantial differences across Europe and the developed world. These different attitudes to provide or retract active care are highly impacted by the ethical, cultural and religious background. Of special concern, the prognosis and estimate of quality of life must be put into the focus of any discussion.

The surprisingly positive self-estimate of affected individuals is supported by the cultural and religious background. Despite the unbridgeable differences between the discussed religions, they all put the right of the handicapped child to live into the focus. They all have in common that discontinuation of active care and palliative care provision represent justifiable alternatives in situations of terminal illness or expected lifelong severe physical, neurological and behavioral impairments as long as religious scripts are interpreted correctly and not utilized to refuse a decision with the notice to a divine trial. Dealing with the parental religious beliefs and correction of any misinterpretation of the religious texts poses a particular challenge to the medical team as was extensively discussed for milk kinship among Muslims (40). On the other hand, physicians need to take care that their counseling gets not directed by the religious background of the parents and the expected parental attitudes. They need to acknowledge that a specific religious background can impact parental decision-making but, nonetheless, individual parents differ in their attitudes toward palliative care. Objective counseling considering all doctoral responsibilities of the Declaration of Geneva and the allowance of a decision process are obligatory (41). The conversation must pay special attention to the parents' needs: unbiased provision of estimates based on latest scientific data have to take into account the parents' needs to be provided information in general terms and as risk percentages. A comprehensive view on the medical situation including the time after the NICU and the possibilities of the parents to participate and to take over responsibility for their child has a major impact on the decision. Counseling of both parents and repeated consultations represent important features. Parental counseling needs to understand and respect the parental perspective, uses a simple and comprehensible language and needs to honor the parental perspectives on medical care. It is important to take into account parental expectations to counseling, which should take place as soon as possible, as well as to address all aspects relevant for their child including visual and written unbiased information and contact data of patient support organizations (42–45). In the decision process, the estimate of the nursing team is at least as relevant as that of the physician. Spiritual support and consultation of the palliative care team can help to reduce the parental burdens (46, 47). Before appealing to a religious council, its competence and responsibility need to be considered by the professional team.

Early and consequent involvement of the multi-professional team and provision of ethical and religious support assist the decision process of the parents and needs to be continued also after decision for therapy retraction. Provision of a comprehensive and fair estimate is the prerogative for the best possible decision. Differences in attitudes to provide active care in newborn infants with severe congenital abnormalities and preterm infants, inter-person differences and moral distress have an important impact on the form of communication and the modalities of withholding or withdrawing active therapy (9, 48, 49). Due to the tremendous improvements in prenatal diagnostics and therapy, comprehensive and repeated counseling of the parents is required already before birth, which paves the way to a basis of trust in critical and emergency situations. Special concern has to be put to allowing the time-consuming decision process, which gets increasingly difficult under economic pressure and in times of shortage of specialists. Comprehensive counseling involving all disciplines, consideration of the ethical, cultural, religious or non-religious, humanistic or agnostic background of the parents, evaluation of familiar resources, emotional pressures and attitudes, and empathetic handling of the parental mourning reaction represent important features to establish a common and stable decision. The aim to reach treatment consent takes center stage during professional counseling. Assumption of the parental view can help in this process. It is important to know that a significant proportion of parents chose to continue life-sustaining therapy after comprehensive professional counseling despite the recommendation to withhold or withdraw active therapy. In contrast to medical standards in the NCIU, there is still a dramatic lack of knowledge on the issues presented in this opinion article. We still need to establish how to best counsel parents and which reactions are provoked by certain actions during parental counseling.

## Author Contributions

FO, TL, and HE initiated and drafted the manuscript. JB, NS, TB, and MB provided valuable input and supervised the manuscript process. RD, TH, HK, UK, YS, and SS drafted the sections dealing with the legal, religious and parental views. SM, H-RT, YB, and K-PZ revised the manuscript. All authors read and approved the final version of the manuscript.

### Conflict of Interest Statement

The authors declare that the research was conducted in the absence of any commercial or financial relationships that could be construed as a potential conflict of interest.
